# Novel Hydrogenated Derivatives of Chemically Modified Curcumin CMC2.24 Are Potent Inhibitors of Melanogenesis in an In Vitro Model: Influence of Degree of Hydrogenation

**DOI:** 10.3390/life13061373

**Published:** 2023-06-12

**Authors:** Shilpi Goenka

**Affiliations:** 1 Department of Biochemistry and Cell Biology, Stony Brook University, Stony Brook, NY 11794-5215, USA; shilp.goenka@gmail.com; 2 Department of Biomedical Engineering, Stony Brook University, Stony Brook, NY 11794-5281, USA

**Keywords:** chemically modified curcumin, CMC2.24, hydrogenation, partial hydrogenation, tetrahydro-CMC2.24, tyrosinase, melanogenesis, melanocytes, skin lightening, recovery

## Abstract

Chemically modified curcumin, CMC2.24, is a promising therapeutic that has shown efficacy in ameliorating excessive pigmentation in our previous studies. However, its inherent disadvantages of color, stability, solubility, and cytotoxicity to melanocytes and keratinocytes at concentrations > 4 µg/mL posed challenges in its use in cosmetic formulations. To overcome these limitations, chemical reduction by hydrogenation of CMC2.24 (compound **1**) was developed to yield products at different time points of hydrogenation (1 h, 2 h, 4 h, and 24 h) referred to as partially (**2**, **3**, **4**) or fully hydrogenated (**5**) products, and the effects of the degree of hydrogenation on melanogenesis in vitro were explored. Compound **1** and products **2**–**5** were evaluated using mushroom tyrosinase activity assays with two substrates (L-tyrosine and L-DOPA), then cellular assays using B16F10 mouse melanoma cells, MNT-1 human melanoma cells, and physiological normal human melanocytes (HEMn-DP cells). The cytotoxicity, melanin contents, cellular tyrosinase activities, and cellular oxidative stress were evaluated. Moreover, the recovery of melanin contents in HEMn-DP cells was also studied. Our results provide novel insights into the role of the degree of hydrogenation of compound **1** on the biological effects of melanogenesis, which were dependent on cell type. To the best of our knowledge, this is the first study to show that in HEMn-DP cells, the anti-melanogenic efficacy of the yellow-colored CMC2.24 is retained as early as 1 h after its hydrogenation; this efficacy is enhanced with longer durations of hydrogenation, with a robust efficacy achieved for the 24 h hydrogenated product **5** at the lowest concentration of 4 µg/mL. A similar potency could be achieved for product **4** at higher concentrations, although interestingly, both differ only by a minor amount of dihydro-CMC2.24. Our results indicate promise for using products **4** & **5** as a skin-lightener in cosmetic formulations with the advantages of lack of color combined with a potency much greater than that of the parent compound **1** at lower concentrations and reversibility of the effects on melanocytes. This, along with the easy synthesis and scale-up of the hydrogenation method for CMC2.24 and the documented higher solubility, stability, and bioavailability of tetrahydrocurcumin, provides further impetus to incorporating these derivatives in cosmetic formulations. The results of this study can help to extend the therapeutic window of the lead compound CMC2.24 by providing options for selecting partially or fully hydrogenated derivatives for cosmetic applications where a trade-off between color and efficacy is needed. Thus, the degree of hydrogenation can be tuned for desired biological effects. Further studies are warranted to evaluate the efficacy of products **4** & **5** at suppressing pigmentation in 3D skin-tissue equivalents and in vivo models.

## 1. Introduction

The overproduction and oversecretion of melanin pigment by specialized cells melanocytes is the causative factor in the occurrence of hyperpigmentation disorders of the skin, which comprise melasma [1], solar lentigines, freckles, diffuse hyperpigmentation [2], periorbital melanosis [3], post-inflammatory hyperpigmentation [4], and drug-induced hyperpigmentation [5]. The current market for treating global pigmentation disorders is projected to reach USD 11.2 billion by 2030 with a cumulative annual growth rate (CAGR) of 6.8% [6]. Based on these reports, it is evident that the demand for novel therapeutics targeted at ameliorating skin pigmentation is rising. 

Chemically modified curcumin (CMC2.24) has emerged as a lead compound that has shown a well-tolerated pharmacokinetic profile and also shown efficacy in in vivo models of diabetes and periodontitis [7,8,9]. Our earlier issued patent [10], which outlined the use of chemically modified curcumins (CMCs) for the inhibition of human melanogenesis, described CMC2.24 as the most potent CMC, including the other two CMCs (CMC2.23 and CMC2.5), with considerable anti-melanogenic activity. Although our previous studies [11,12] reported that CMC2.24 (lead compound **1**) was the most potent inhibitor of melanogenesis, its cytotoxicity to keratinocytes at the lowest concentration of 4 µg/mL and to melanocytes at concentrations > 4 µg/mL limited the span of its concentration ranges. In addition, the yellow coloration of CMC2.24 (similar to parent curcumin) hindered its incorporation in cosmetic formulations for commercial development due to the compromise of the aesthetic appeal of the formulations. 

Hence, attempts to hydrogenate (to eliminate color and enhance stability and bioavailability) were carried out for all these three CMCs. In earlier preliminary experiments (unpublished observations), we obtained purified samples of tetrahydro derivatives of CMC2.24 and CMC2.5, referred to as TH-CMC2.24 and TH-CMC2.5, and conducted a preliminary evaluation of their effects on melanogenesis, where TH-CMC2.24 showed the capacity to inhibit melanogenesis, while TH-CMC2.5 did not have any effect. Notably, TH-CMC2.23 was not evaluated due to difficulties in one or more synthesis steps of hydrogenation and was thus abandoned. The initial encouraging results of TH-CMC2.24 prompted us to undertake a commercial hydrogenation synthesis involving hydrogenation of the parent compound CMC2.24 similar to the methods based on previous reports [13,14] and patent [15] that synthesized tetrahydrocurcumin (THC) from the catalytic hydrogenation of curcumin. However, in a previous report [16] where the authors studied the kinetics of hydrogenation of curcumin, it was shown that molecules with distinct structures could be formed during the hydrogenation reaction. During this course, the idea was considered that the anti-melanogenic activity of CMC2.24 might depend on the degree of hydrogenation due to the formation of distinct intermediates during chemical reduction. Hence, we obtained different compounds at different time points of hydrogenation, referred to as partially or fully hydrogenated products of CMC2.24. 

In this study, we have, for the first time, evaluated the biological activities of partially and fully hydrogenated CMC2.24 derivatives and studied the effects of the degree of hydrogenation on melanogenesis. The results obtained from this study would help us to understand better how different structural modifications on the skeleton of curcumin can have a role in modulating the effects on pigmentation, thereby helping to identify structural moiety and the optimum time of hydrogenation reaction to yield derivatives which can be acceptable for cosmetic use. The results of this study are significant as they provide a proof of principle that can help in extending the therapeutic window of the use of the lead compound CMC2.24 by providing more options for the selection and use of partially or fully hydrogenated derivatives for cosmetic applications and other applications where a trade-off between color and efficacy may be needed. 

## 2. Materials and Methods

### 2.1. Materials

CMC2.24 (compound **1**) was kindly provided by Traverse Biosciences Inc. (Stony Brook, NY, USA). Mushroom tyrosinase, L-Tyrosine, and L-DOPA were procured from Sigma-Aldrich (St. Louis, MO, USA). Hanks’ balanced salt solution (HBSS), Dulbecco’s modified Eagle’s medium (DMEM), phosphate-buffered saline (PBS), TrypLE Express (1×), and penicillin-streptomycin were purchased from Thermo Fisher Scientific (Waltham, MA, USA). MTS reagent (CellTiter 96^®^ AQueous One Solution) was purchased from Promega Corporation (Madison, WI, USA). AIM-V and Minimum essential medium (MEM) were procured from Gibco (Thermo Fisher Scientific). Heat-inactivated fetal bovine serum (HI-FBS) was obtained from R&D Systems Inc. (Minneapolis, MN, USA). Cell-lysis buffer was obtained from Signosis Inc. (Santa Clara, CA, USA).

### 2.2. Hydrogenation of CMC2.24

The hydrogenation of curcumin using a 10% palladium (Pd)/carbon catalyst to synthesize THC has been reported previously [13,14,15]. Hence, partially and fully hydrogenated CMC2.24 (products **2**, **3**, **4**, **5**) were synthesized using a similar hydrogenation method with some modifications. The chemical synthesis was carried out at Sterling Pharma Solutions (Cary, NC, USA) under the aegis of Biocogent LLC (Stony Brook, NY, USA). Briefly, 2 g of CMC2.24 (compound **1**) was added in ethyl acetate (200 mL) and stirred for 30 min, followed by the addition of 10% Pd/C (250 mg), and kept for hydrogenation on a parr-shaker at 50 PSI for 1 h. After this step, the reaction mass was filtered on a nylon filter (0.45 microns) and washed with ethyl acetate (50 mL). The filtrate color did not change, and it was concentrated at a bath temperature of 32 °C to yield a yellow solid (product **2**). To obtain the other partially hydrogenated products **3**, **4**, and fully hydrogenated product **5**, the hydrogenation was conducted for 2 h, 4 h, and 24 h, respectively. Samples of the four hydrogenated products were analyzed by proton nuclear magnetic resonance (1H-NMR) spectroscopy (Figures S1–S4), similar to a previous study of curcumin analogs [17].

### 2.3. Compositional Analysis by Mass Spectrometry

Samples were analyzed using a Thermo LTQ Ion-trap Mass Spectrometer (MS; Thermo-Fisher, Waltham, MA, USA) using a Luna^®^ C-18 HPLC column with a mobile phase containing an acetonitrile–water phase. Selected ion monitoring (SIM) of [M-H]- with an *m*/*z* ± 0.5 Da of compounds was used with a 200 µL/minute flow rate. The HPLC eluted the compounds into the mass spectrometer running in the negative ion mode under a voltage of 3.5 kV and capillary of 35 V, and the peaks were identified. Their areas were calculated to determine the percentage of each molecule in the hydrogenated compounds. The SIM chromatograms of each product are listed under supplementary material (Figures S5–S8).

### 2.4. Mushroom Tyrosinase Activity

The monophenolase and diphenolase activity assays were conducted similarly to the methods described in our earlier study [18]. All the test samples (**1**–**5**) were prepared at 2.5× concentrations in 100% DMSO, and 80 µL of each compound was combined with 100 µL of 2 mM L-tyrosine substrate solution in a 96-well microplate. The reaction was commenced by adding 20 µL of 12.5 µg/mL mushroom tyrosinase, and the absorbance of dopachrome formation was measured at 475 nm using the kinetic mode for 20 min. The tyrosinase activities were determined from the slopes of the linear range of the progress curves of the reaction and expressed as a percentage of the untreated control. 

A similar method was used to estimate the effects of compound **1** and products **2**–**5** on diphenolase activity, except the substrate used was 100 µL of 6 mM L-DOPA and 20 µL of the enzyme (at a final concentration of 3.5 µg/mL) was used. The slopes of the kinetic readings were calculated to determine and compare tyrosinase activity from the untreated control.

### 2.5. Cell Culture

B16F10 cells were obtained from American Type Culture Collection (ATCC; Manassas, VA, USA), and HaCaT cells were procured from AddexBio (San Diego, CA, USA). Both cells were cultured using DMEM supplemented with 10% HI-FBS and 1% penicillin-streptomycin antibiotics. MNT-1 human melanoma cells were kindly provided by Dr. Michael Marks (University of Pennsylvania, Philadelphia, PA, USA) and were maintained using DMEM supplemented with 18% HI-FBS, 10% AIM-V medium, 1% MEM, and 1% antibiotics. HEMn-DP cells were procured from Cascade Biologics (Portland, OR, USA) and cultured in medium 254 supplemented with 1% human melanocyte growth supplement (HMGS; Cascade Biologics) and 1% antibiotics. All the cells were maintained in a humidified incubator with 95% air—5% CO_2_ at 37 °C.

### 2.6. MTS Cytotoxicity Assay

#### 2.6.1. MTS Cytotoxicity Assay in B16F10 Cells 

B16F10 cells (0.8 × 10^4^ cells/well in 200 µL medium) were seeded in 200 µL complete medium in a 96-well plate for 24 h. After this, varying concentrations of the test samples (**1**, **2**, **3,** **4**, **5**) were added to a 200 µL medium. The control group was treated with 0.25% DMSO (solvent control), and the cultures were maintained for 3 d. Subsequently, the cell viability was estimated by the MTS assay based on the manufacturer’s instructions. Briefly, the culture medium was aspirated, 100 µL of complete medium containing 20 µL of MTS reagent was added, and the plate was incubated for 1 h at 37 °C. After this, 100 µL were aliquoted in a new 96-well plate, and the absorbance was read at 490 nm. Cell viability was reported as a percentage of the untreated control.

#### 2.6.2. MTS Cytotoxicity Assay in MNT-1 Cells 

For 24 h, 1.2 × 10^4^ MNT-1 cells were plated in 200 µL complete medium in a 96-well plate, after which, the compound **1** and products **2**–**5** were added to 200 µL medium, while the control group was treated with 0.25% DMSO (solvent–control). The cultures were maintained for 5 d (with samples renewed on the third day). After 5 d, cell viability was assessed using an MTS assay, similar to the method reported above.

#### 2.6.3. MTS Cytotoxicity Assay in HaCaT Cells 

For 24 h, 0.7 × 10^4^ HaCaT cells were plated in 200 µL complete medium in a 96-well plate, after which, the compounds were added, and cultures were continued for 5 d, with compound **1** and products **2**–**5** renewed on the third day of culture. At the end of 5 d, the cell viability was assessed using an MTS assay, similar to the method reported above.

#### 2.6.4. MTS Cytotoxicity Assay in HEMn-DP Cells 

For 48 h, 0.85 × 10^4^ HEMn-DP cells were plated in 200 µL complete medium in a 96-well plate, after which, compound **1** and products **2**–**5** were added, and cultures were maintained for 6 d, with compounds renewed on the third day of culture. At the end of 6 d, the MTS assay was conducted similarly to the abovementioned method, except an incubation time of 2 h was used.

### 2.7. Cellular Melanin Assay

#### 2.7.1. Intracellular Melanin Assay in B16F10 Cells 

B16F10 cells (6 × 10^4^ cells/well in 1.5 mL complete medium) were seeded in a 12-well plate for 24 h, after which, compound **1** and products **2**–**5** were added in 1.5 mL medium at various nontoxic concentrations. At the same time, the control group was treated with 0.25% DMSO (solvent–control), and cultures were maintained for a duration of 3 d. After this, cells were detached and pelleted, then washed in PBS, 200 µL of 1N NaOH was added, and tubes were heated in a water bath at 75 °C to solubilize intracellular melanin. Next, 150 µL of lysates were transferred to a 96-well plate, and the absorbance was read at 475 nm; this value was normalized to the total protein content (calculated from a portion of lysate after centrifugation). Abs/µg protein was expressed as a percentage of the solvent control to obtain relative melanin levels of each treatment group.

#### 2.7.2. Intracellular Melanin Assay in MNT-1 Cells 

For 24 h, 1.65 × 10^5^ MNT-1 cells were seeded in 12-well plates, after which, the compounds were added, and the cultures were maintained for 5 d, with compound **1** and products **2**–**5** replenished once during the culture. After this, cells were detached and pelleted, then washed in PBS, and then 250 µL of 1N NaOH was added and heated at 75 °C. Next, 200 µL of lysates were transferred to a 96-well plate, and the melanin levels were determined similarly to the method described in Section 2.7.1. 

#### 2.7.3. Intracellular Melanin Assay in HEMn-DP Cells 

HEMn-DP cells were seeded in 6-well plates and cultured for 4 d, after which, the culture medium was replaced with compound **1** and products **2**–**5**, and the cultures were maintained for 6 d (with samples renewed once during the maintenance period). After this exposure, the cell pellets were inspected for color alterations, and the melanin levels were determined by a hot NaOH lysis method similar to that described in Section 2.7.1. 

### 2.8. Tyrosinase Activity Assay in HEMn-DP Cells

HEMn-DP cells were seeded in 12-well plates and cultured for 3 d, after which, compound **1** and products **2**–**5** were added, and the cultures were maintained for 6 d. At the end of treatments, the cells were harvested and lysed; lysates were centrifuged, and the supernatants were aliquoted in a 96-well plate, followed by the addition of a freshly prepared solution of L-DOPA substrate; the absorbance was measured at 475 nm in the kinetic mode for a period of 20 min, and the slopes of the linear range of the velocities were used to determine the tyrosinase activity after normalization to total protein contents.

### 2.9. Reactive Oxygen Species (ROS) Generation in HEMn-DP Cells

The method for the estimation of ROS was similar to the one reported in our previous study [19]. Briefly, HEMn-DP cells were cultured in 24-well plates and, after 3 d, were treated with compound **1** and products **2**–**5**, and the cultures were maintained for 6 d. At this point, the cells were washed in HBSS, and 25 µM of the DCFDA probe was added and incubated at 37 °C for 30 min. After this, cells were lysed, centrifuged, and aliquoted in a black 96-well plate. The fluorescence was read at 485/535 nm wavelength in a Gemini Microplate reader. The relative fluorescence units (RFU) values were normalized to the total protein content absorbances and reported as % of control.

### 2.10. Recovery Experiments in HEMn-DP Cells

An exposure recovery study was conducted to assess if the effects of compound **1** and products **2**–**5** on melanogenesis in HEMn-DP cells can be reversed upon the removal of compounds from the culture medium. Briefly, DP cells were treated with compounds for a duration of 6 d, after which the melanin assay was conducted similarly to the previous method. Another set of cultures was washed with HBSS and was continued for an additional 7 d in fresh culture medium without the samples (with one medium renewal in between). The cultures were processed at the end of the 7 d recovery period to estimate melanin content. Data are reported as relative melanin levels expressed as a percentage of the control after normalization to total protein contents.

### 2.11. Statistical Analysis

All data are expressed as mean ± SD and analyzed by one-way analysis of variance (ANOVA) with Dunnett’s post hoc test using GraphPad Prism software (version 8.4.0, San Diego, CA, USA). The level of statistical significance was considered at a level of *p* < 0.05.

## 3. Results

### 3.1. Compositional Estimation of the Hydrogenated Products

**Figure 1:**
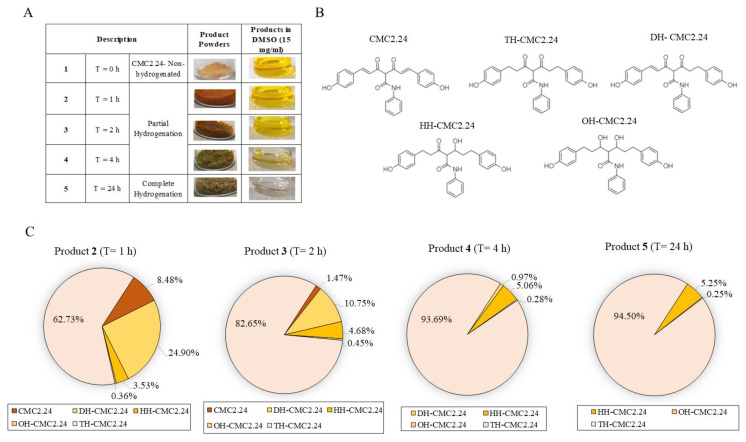
(**A**) Hydrogenated and partially hydrogenated products of CMC2.24 (compound **1**) with the visible gradation in the intense yellow coloration of mixtures in solution form (1.5%); (**B**) chemical structures of compound **1** and its hydrogenated metabolites (Tetrahydro CMC2.24; TH-CMC2.24, Dihydro CMC2.24; DH-CMC2.24, Hexahydro CMC2.24; HH-CMC2.24, and Octahydro CMC2.24; OH-CMC2.24); (**C**) Pie charts showing hydrogenated products **2**, **3**, **4**, and **5** with their compositional analysis.

The photos of the samples of the parent compound **1** and the four products obtained from hydrogenation at different time points of 1 h, 2 h, 4 h, and 24 h, with their reconstituted solution in DMSO (1.5%), are depicted in Figure 1A. The different hydrogenation reactions yielded distinct mixtures of curcumin derivatives, referred to as ‘products’ in the whole manuscript for consistency. The diminution of the intense yellow color of lead compound **1** is evident as the hydrogenation time progressed; product **5** was considered fully hydrogenated, while products **2**, **3**, and **4** were referred to as partially hydrogenated compounds. The chemical structures of different molecules that comprise the hydrogenated mixture are shown in Figure 1B and consist of Tetrahydro CMC2.24 (TH-CMC2.24), dihydro CMC2.24 (DH-CMC2.24), hexahydro CMC2.24 (HH-CMC2.24), and octahydro CMC2.24 (OH-CMC2.24). 

The analysis of the compositions of the four hydrogenated products is summarized in Figure 1C. The results show that products **2** and **3** retain residual amounts of CMC2.24, which underwent complete hydrogenation at time points of 4 h onwards. Although products **4** and **5** have remarkably close amounts of TH-CMC2.24, they differ by the amount of DH-CMC2.24 (1%). Product **5** has no dihydro CMC2.24 left, indicative of complete hydrogenation.

### 3.2. Effects on Cell-Free Tyrosinase Activity

**Figure 2:**
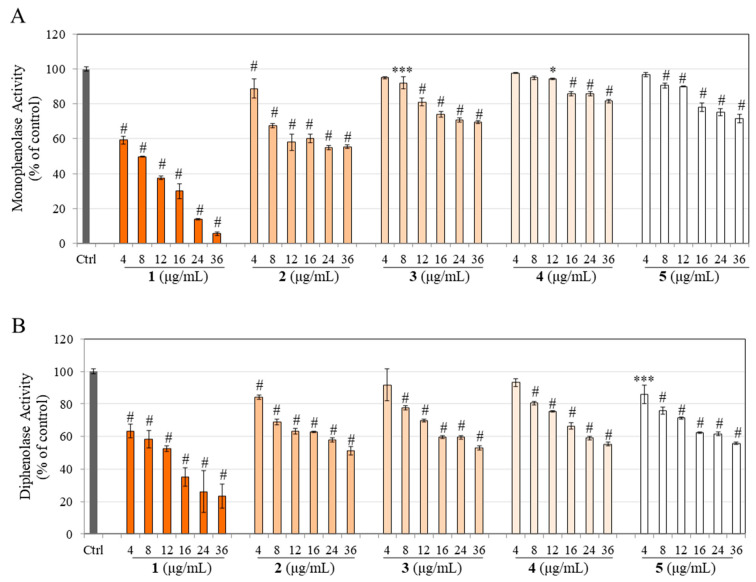
Effect of parent CMC2.24 (**1**) and its hydrogenated products **2**, **3**, **4**, and **5** on the activity of mushroom tyrosinase catalyzed by (**A**) L-Tyrosine (monophenolase) substrate or by (**B**) L-DOPA (diphenolase) substrate. (* *p* < 0.05, *** *p* < 0.001, and # *p* < 0.0001 vs. Ctrl), All data are mean ± SD of triplicate determinations.

Our initial screening comprised cell-free assays to examine whether the hydrogenated derivatives of CMC2.24 (compound **1**) might retain the tyrosinase inhibitory potential of compound **1**. Our results on monophenolase activity (equivalent to the first step of tyrosinase-catalyzed reaction) revealed that compound **1** robustly inhibited the monophenolase activity by 40.82%, 50.35%, 62.46%, 70.02%, 86.26%, and 94.57% at 4, 8, 12, 16, 24, and 36 µg/mL concentrations, respectively. In comparison, product **2** significantly inhibited monophenolase activity by 11.14%, 32.51%, 41.93%, 39.88%, 45.18%, and 44.51% at 4, 8, 12, 16, 24, and 36 µg/mL concentrations, respectively (Figure 2A). Product **3** significantly inhibited monophenolase activity by 7.90%, 18.84%, 25.93%, 29.25%, and 30.46% at 8, 12, 16, 24, and 36 µg/mL concentrations, respectively, while product **4** significantly inhibited monophenolase activity by 5.43%, 14.22%, 14.22%, and 18.24% at 12, 16, 24, and 36 µg/mL concentrations, respectively (Figure 2A). Lastly, product **5** significantly inhibited monophenolase activity by 9.40%, 9.87%, 21.89%, 24.62%, and 28.32% at 8, 12, 16, 24, and 36 µg/mL concentrations, respectively (Figure 2A). 

Overall, these results show that compared to compound **1,** which was expectedly a potent inhibitor of monophenolase activity, the partially hydrogenated products **2** and **3** showed a differential inhibitory profile compared to **4** and **5**. In contrast, products **4** and **5** retained moderate inhibitory effects at concentrations ≥ 16 µg/mL, although the effects were lost at a lower concentration of 4 µg/mL. The potency of monophenolase inhibition followed the order: **1** >> **2** > **3** > **5** > **4**.

We next estimated the diphenolase activity (equivalent to the second step of the tyrosinase-catalyzed reaction); results showed that compound **1** significantly inhibited diphenolase activity by 36.68%, 41.75%, 47.50%, 64.90%, 73.85%, and 76.65% at 4, 8, 12, 16, 24, and 36 µg/mL concentrations, respectively, while product **2** significantly inhibited diphenolase activity by 15.91%, 31.08%, 37.03%, 37.21%, 42.29%, and 48.49% at 4, 8, 12, 16, 24, and 36 µg/mL concentrations, respectively (Figure 2B). Next, product **3** significantly inhibited diphenolase activity by 22.50%, 30.27%, 40.40%, 40.56%, and 46.87% at 8, 12, 16, 24, and 36 µg/mL concentrations, respectively, while product **4** significantly inhibited diphenolase activity by 19.49%, 24.56%, 33.61%, 40.92%, and 44.52% at 8, 12, 16, 24, and 36 µg/mL concentrations, respectively (Figure 2B). Lastly, product **5** significantly inhibited diphenolase activity by 14.13%, 24.34%, 28.67%, 37.76%, 38.42%, and 44.15% at 4, 8, 12, 16, 24, and 36 µg/mL concentrations, respectively (Figure 2B).

Overall, these results show that all the partially and fully hydrogenated products retained diphenolase inhibitory potencies of the parent compound in a concentration-dependent manner. Compared to the partially hydrogenated products (**2**, **3**, **4**), product **2** was more potent than **3** and **4**. The potency of diphenolase inhibition followed the order: **1** >> **2** > **5** > **3** = **4**.

### 3.3. Effects on Viability and Melanogenesis in B16F10 Mouse Melanoma Cells

**Figure 3:**
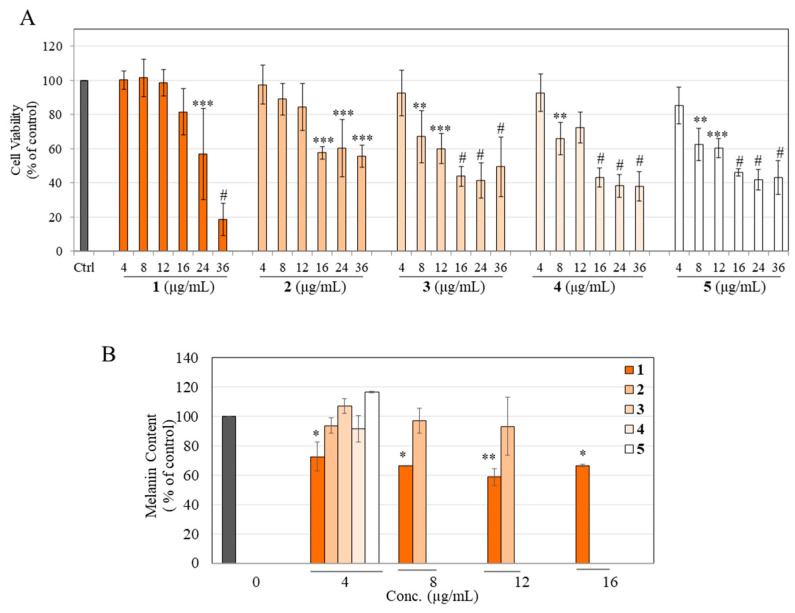
(**A**) Cytotoxicity of parent CMC2.24 (**1**) with its partially hydrogenated (**2**,**3**,**4**) and fully hydrogenated (**5**) products in B16F10 mouse melanoma cells after a 3 d treatment; (**B**) Melanin content of B16F10 cells treated with nontoxic concentrations of compound **1** and hydrogenated products **2**, **3**, **4**, and **5** for 3 d (one-way ANOVA with Dunnett’s test; * *p* < 0.05, ** *p* < 0.01, *** *p* < 0.001 and # *p* < 0.0001 vs. control group). Data for (**A**) are the mean ± SD of at least three independent experiments, while data for (**B**) are the mean ± SD of two independent experiments.

Our results showed that compound **1** displayed significant cytotoxicity to B16F10 cells at higher concentrations of 24 and 36 µg/mL, while product **2** displayed significant cytotoxicity at 16, 24, and 36 µg/mL, respectively (Figure 3A). Products **3**, **4**, and **5** exhibited greater cytotoxicity and significantly suppressed viability at all concentrations ≥ 8 µg/mL (Figure 3A). The cytotoxicity to B16F10 cells followed the order: **3** = **5** > **4** >> **2** > **1**. 

For melanogenesis experiments, the nontoxic concentration of compound **1** and products **2**–**5** was used. Unexpectedly, the results showed that the partially hydrogenated products **2**, **3**, and **4**, and the fully hydrogenated product **5** did not affect the intracellular melanin contents, while the parent compound **1**, as expected, significantly diminished melanin content by 27.33%, 33.50%, 41.25%, and 33.47%, at concentrations of 4, 8, 12, and 16 µg/mL, respectively (Figure 3B).

### 3.4. Effects on Viability and Melanogenesis in MNT-1 Human Melanoma Cells

**Figure 4:**
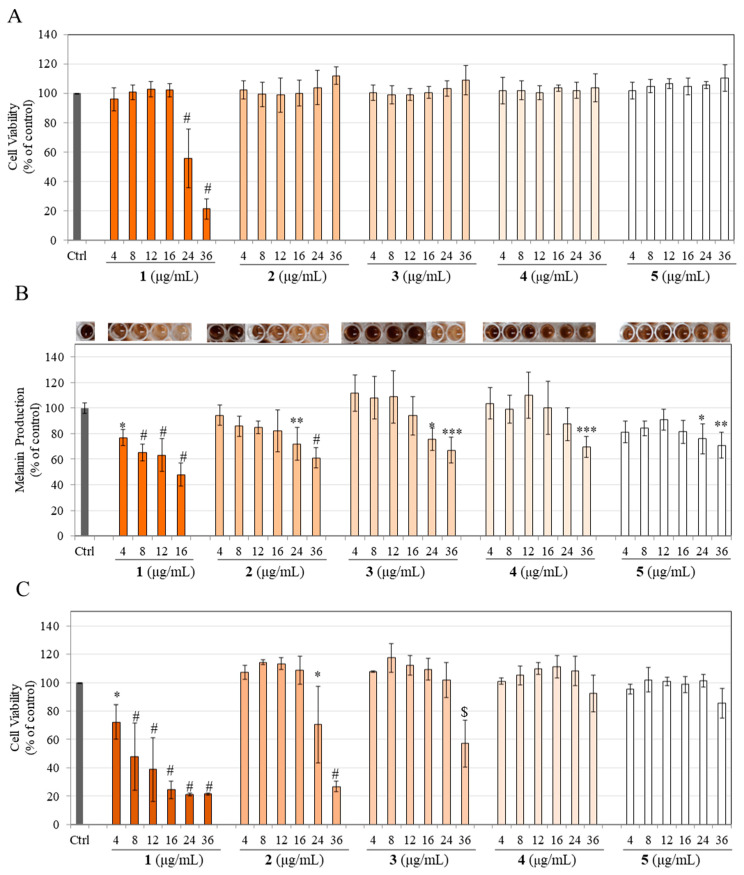
(**A**) Cytotoxicity assay of compound **1** and hydrogenated products **2**, **3**, **4**, and **5** in MNT-1 human melanoma cells after 5 d treatments; # *p* < 0.0001 vs. control group by one-way ANOVA with Dunnett’s test; data are mean ± SD of at least three independent experiments; (**B**) Spectrophotometric estimation of intracellular melanin in MNT-1 cells treated with compound **1** and products **2**, **3**, **4**, and **5** for 5 d, with the corresponding panel showing representative photos of lysates of cell pellets (one-way ANOVA with Dunnett’s test, * *p* < 0.05; ** *p* < 0.01, *** *p* < 0.001 and # *p* < 0.0001 vs. control group). Data are mean ± SD of values combined from at least three independent experiments, each run in duplicate; (**C**) Cytotoxicity assay of compound **1** and products **2**, **3**, **4**, and **5** in HaCaT cells after a 5 d treatment. One-way ANOVA with Dunnett’s test, * *p* < 0.05; $ *p* < 0.001 and # *p* < 0.0001 vs. control group. Data are mean ± SD of at least three independent experiments.

Our results showed that parent compound **1** was nontoxic at 4, 8, 12, and 16 µg/mL concentrations but significantly lowered MNT-1 cell viability to 55.45% and 21.21% at 24 and 36 µg/mL, respectively (Figure 4A). At the same time, the partially hydrogenated products (**2**, **3**, **4**) and fully hydrogenated product **5** were nontoxic over the entire concentration range (Figure 4A). Based on these data, nontoxic concentrations of compound **1** and products **2**–**5** were evaluated in the melanogenesis experiments. 

The color of the cell lysates aliquoted in the wells before quantitation was darkest for the control group, with a visible reduction in coloration in a concentration-dependent manner for various samples (Figure 4B, photos). Quantitation of relative melanin contents revealed that the parent compound **1** significantly diminished melanin content in a concentration-dependent manner by 23.10%, 34.88%, 36.77%, and 52.22%, at concentrations of 4, 8, 12, and 16 µg/mL, respectively (Figure 4B). Product **2** significantly diminished melanin contents by 28.05% and 38.94% at concentrations of 24 and 36 µg/mL, respectively, while product **3** significantly diminished melanin content by 24.62% and 32.94% at concentrations of 24 and 36 µg/mL, respectively (Figure 4B). Product **4** showed a significant diminution of melanin content by 30.59% at the highest concentration of 36 µg/mL, while product **5** showed significant diminutions of 23.99% and 29.17% at 24 and 36 µg/mL, respectively (Figure 4B). 

These results show that the degrees of melanogenesis inhibition differed among all derivatives, with parent compound **1** being the most potent suppressor of melanin production in MNT-1 human melanoma cells. Nonetheless, the inhibitory potency of the parent compound **1** at 4 µg/mL was still retained in the partially hydrogenated products **2** and **3** and fully hydrogenated product **5** at a 6-fold higher concentration, while it was retained by partially hydrogenated product **4** at a 9-fold higher concentration, which was an exciting finding (Figure 4B). 

### 3.5. Effects on the Viability of Human Keratinocytes

To test the safety of the samples for topical use, their cytotoxicity was examined in HaCaT cells, which are immortalized human keratinocytes. Results showed that compound **1** significantly lowered cell viability to 72.31%, 47.83%, 38.70%, 24.38%, 21.22%, and 21.40% at concentrations of 4, 8, 12, 16, 24, and 36 µg/mL, respectively (Figure 4C). Product **2** significantly lowered cell viability to 70.40% and 26.78% at concentrations of 24 and 36 µg/mL, respectively, while product **3** significantly lowered cell viability to 57.06% at the highest concentration of 36 µg/mL (Figure 4C). Lastly, products **4** and **5** were nontoxic over the entire concentration range of 4–36 µg/mL (Figure 4C). The cytotoxicity profile followed the order: **1** >> **2** > **3** > **4** = **5**. 

### 3.6. Effects on Cellular Viability in Primary Human Melanocytes from Darkly Pigmented Skin (HEMn-DP)

**Figure 5:**
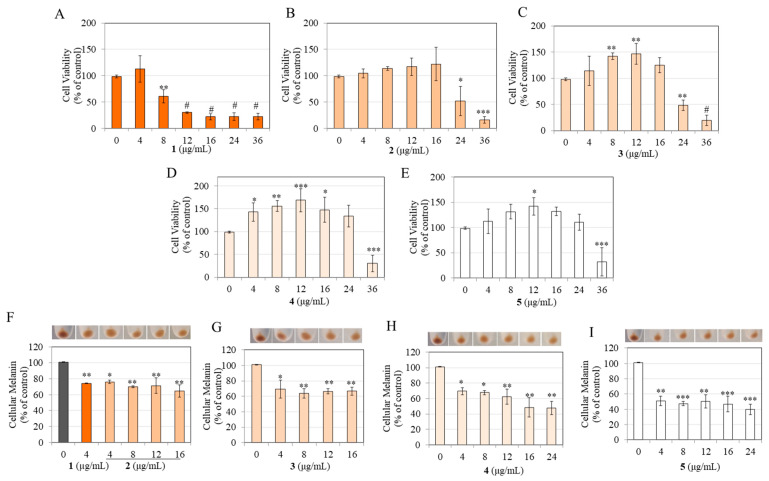
Cytotoxicity of HEMn-DP cells treated with (**A**) compound **1**, (**B**) product **2**, (**C**) product **3**, (**D**) product **4**, and (**E**) product **5** for a period of 6 d. Spectrophotometric determination of cellular melanin levels with corresponding photos of pellets of HEMn-DP cells treated with (**F**) compound **1** and product **2**, (**G**) product **3**, (**H**) product **4**, and (**I**) product **5** (* *p* < 0.05, ** *p* < 0.01, *** *p* < 0.001, and # *p* < 0.0001 vs. control group; one-way ANOVA with Dunnett’s test). Data for (**A**–**C**) are mean ± SD of at least three independent experiments, for (**D**,**E**) is mean ± SD of four independent experiments, and for (**F**,**G**) is mean ± SD of at least two independent experiments.

The samples were next assayed in normal human melanocytes obtained from darkly pigmented skin, which is more physiological than human melanoma cells. Our results showed that compound **1** induced the greatest cytotoxicity to HEMn-DP cells with a significant suppression in viability by 37.36%, 68.10%, 75.96%, 76.01%, and 75.74% at 8, 12, 16, 24, and 36 µg/mL concentrations, respectively (Figure 5A). Product **2** significantly suppressed cell viability by 46.68% and 82.16% at 24 and 36 µg/mL concentrations, respectively (Figure 5B), while product **3** showed a similar cytotoxicity profile with significant suppression in viability by 50.15% and 78.53% at 24 and 36 µg/mL concentration, respectively (Figure 5C). Products **4** and **5** were the least cytotoxic, with a significant diminution in viability by 68.77% (product **4**) and 66.83% (product **5**) only at the highest concentration of 36 µg/mL (Figure 5D,E). Taken together, the profile of cytotoxicity in HEMn-DP cells followed the order: **1** >> **2** = **3** > **4** = **5**. 

Based on these results, we selected compound **1** at 4 µg/mL, products **2** and **3** over the 4–16 µg/mL concentration range, and products **4** and **5** over the 4–24 µg/mL concentration range for subsequent experiments.

### 3.7. Effects on Melanin Production in HEMn-DP Cells

The photos of human melanocytes after treatment with various samples were visibly lighter than the control group; this effect was more pronounced for products **4** and **5** (Figure 5F–I, photo panel). Compound **1** significantly diminished melanin levels in HEMn-DP cells by 26.69% at 4 µg/mL (Figure 5F). Encouragingly, the partially hydrogenated product **2** continued to retain this inhibitory efficacy of parent compound **1** at 4 µg/mL with a concentration-dependent diminution; significant inhibitions of 25.02%, 31.04%, 29.83%, and 36.50% were obtained for product **2** at concentrations of 4, 8, 12, and 16 µg/mL, respectively (Figure 5F). Product **3** also showed a similar inhibitory profile as product **2,** with significant diminution in melanin levels by 31.92%, 37.21%, 34.47%, and 34.39%, at concentrations of 4, 8, 12, and 16 µg/mL, respectively (Figure 5G). Product **4** continued to show a similar inhibitory profile to product **3** with significant diminution in melanin levels by 31.48%, 33.38%, and 38.89% at 4, 8, and 12 µg/mL, respectively (Figure 5H). However, a greater diminution was obtained at higher concentrations; the levels were significantly suppressed by 52.53% and 53.50% at the concentration of 16 and 24 µg/mL, respectively (Figure 5H). Lastly, our results of product **5** showed the most potent efficacy as the levels of melanin were significantly diminished by 50.05%, 53.68%, 50.98%, 54.17%, and 61.02% at concentrations of 4, 8, 12, 16, and 24 µg/mL, respectively (Figure 5I). 

Collectively, our results demonstrate that the inhibitory efficacy of parent compound **1** on melanin levels in HEMn-DP cells was dependent on the degree of hydrogenation; complete hydrogenation (24 h) was needed to obtain a color-free product that was also 1.87-fold more potent in diminishing melanin production at the lowest concentration of 4 µg/mL.

**Figure 6:**
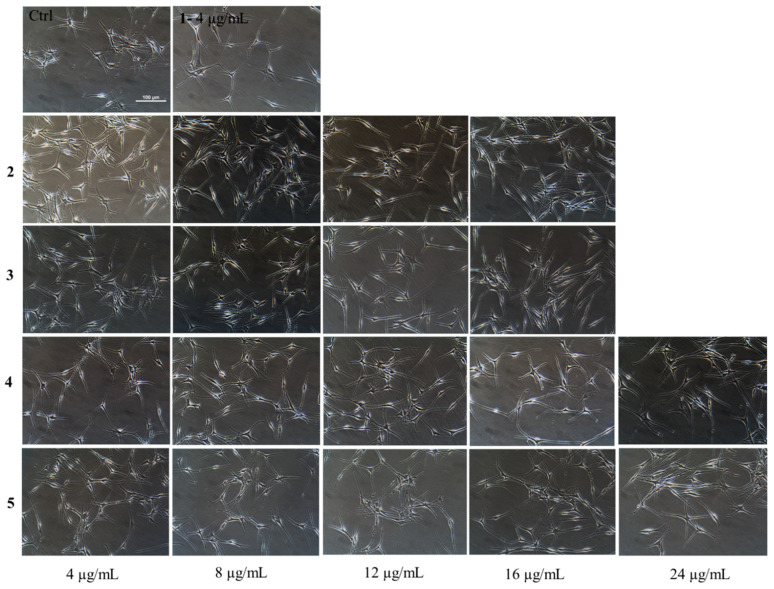
Representative phase-contrast images of HEMn-DP cells of the untreated control group and treatment groups for compound **1** at a concentration of 4 µg/mL and products **2**, **3**, **4,** and **5** over 4–24 µg/mL concentration range.

We also examined the morphology of HEMn-DP cells after exposure to compound **1** and products **2**–**5**. A qualitative examination of images revealed that while the cells in the untreated control group possessed a multidendritic morphology, the cells treated with compound **1** at 4 µg/mL showed less dendritic appearance; this effect was enhanced at higher concentrations for the hydrogenated products, especially at 16 µg/mL where all the products seemed to have a bi- or tri-dendritic morphology (Figure 6). 

### 3.8. Effects on Tyrosinase Activity in HEMn-DP Cells

**Figure 7:**
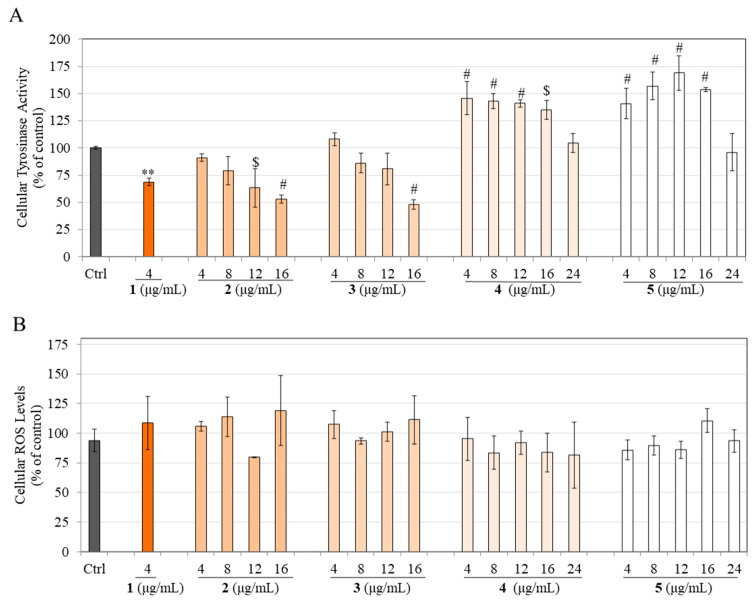
(**A**) Tyrosinase activity and (**B**) ROS levels measured in lysates of HEMn-DP cells treated with compound **1** and products **2**, **3**, **4**, and **5** for 6 d; one-way ANOVA with Dunnett’s test. ** *p* < 0.01, $ *p* < 0.001, # *p* < 0.0001 vs. control group. All data are mean ± SD of values combined from at least two independent experiments.

As all the hydrogenated products were shown to diminish melanin production in HEMn-DP cells, we next examined if that might be correlated, at least in part, to an inhibition of the activity of the enzyme tyrosinase. Our results showed that compound **1** at 4 µg/mL significantly inhibited cellular tyrosinase activity by 31.24%. In contrast, product **2** significantly inhibited cellular tyrosinase activity by 36.69% and 47.14% but only at higher concentrations of 12 and 16 µg/mL, respectively (Figure 7A). Product **3** significantly inhibited tyrosinase activity by 52.10% at the highest concentration of 16 µg/mL with no effect at lower concentrations (Figure 7A). 

Intriguingly, products **4** and **5** were shown to have the opposite effect and stimulated tyrosinase activity over the concentration range of 4–16 µg/mL with no effect at the highest concentration of 24 µg/mL (Figure 7A). Product **4** significantly stimulated tyrosinase activity by 45.80%, 43.21%, 40.91%, and 35.06% at concentrations of 4, 8, 12, and 16 µg/mL, respectively, and product **5** significantly stimulated tyrosinase activity by 40.85%, 56.98%, 69.03%, and 53.62% at concentrations of 4, 8, 12, and 16 µg/mL, respectively. Altogether, these results indicate that the anti-melanogenic mechanisms of products **4** and **5** are unrelated to their effects on tyrosinase activity and might involve other mechanisms.

### 3.9. Effects on Intracellular ROS Levels in HEMn-DP Cells

Our results showed that after exposure to compound **1** and products **2**–**5**, the levels of intracellular ROS were unchanged and showed no statistical difference compared to the untreated control (Figure 7B).

### 3.10. Study of the Reversibility of Inhibitory Effects on Melanin Production in HEMn-DP Cells

**Figure 8:**
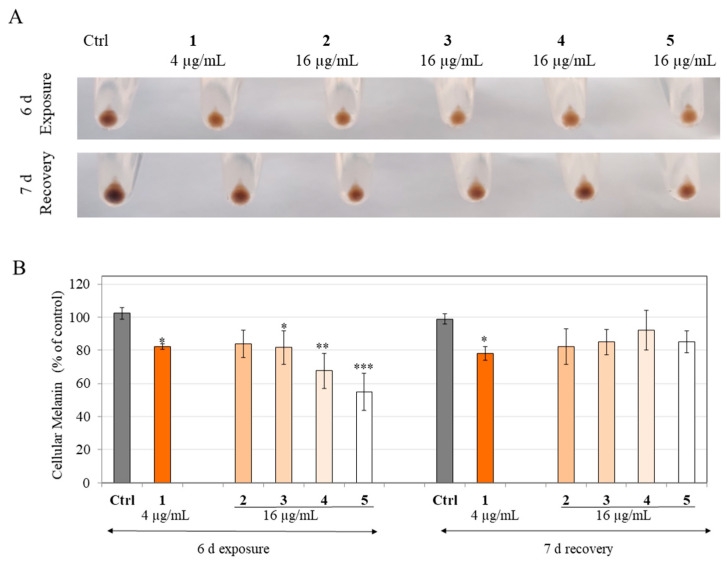
Recovery study of melanogenesis by compound **1** and products **2**–**5** in HEMn-DP cells. (**A**) Representative photos of HEMn-DP cell pellets for exposure-recovery and (**B**) Intracellular melanin level in HEMn-DP cells treated with compound **1** at 4 µg/mL and products **2**–**5** at 16 µg/mL for a period of 6 d exposure and 7 d recovery. All data are mean ± SD of at least three independent experiments. * *p* < 0.05, ** *p* < 0.01, and *** *p* < 0.001 vs. Ctrl; one-way ANOVA with Dunnett’s test.

Our results showed that the pellets of HEMn-DP cells treated with compound **1** at 4 µg/mL and products **2**–**5** at 16 µg/mL for a period of 6 d showed a lighter color on visual inspection (Figure 8A, exposure panel) for all samples with the lightest colored pellet for product **5**. Quantitation by the hot NaOH lysis method showed that compound **1** (4 µg/mL) inhibited melanin levels by 20.02%. In contrast, the levels of inhibition were 18.45%, 20.56%, 34.63%, and 47.34% for products **2**, **3**, **4**, **5** that were tested at 16 µg/mL, respectively (Figure 8B). 

After the cultures of HEMn-DP cells were continued in the sample-free medium for a further 7d (recovery period), the cell pellets were again inspected (Figure 8A, recovery panel). Our results on melanin levels in cells showed that for cells treated with compound **1,** melanin levels were still significantly lower than the recovery control group by 20.82%, while for cells treated with products **2**, **3**, **4**, and **5**, the melanin levels were almost recovered and were not statistically different from that of the recovery control group (Figure 8B). Noteworthily, recoveries of 27.86% and 33.47% from the inhibited percentages were achieved for product **4** and product **5**, respectively, which is remarkable, especially since these products diminished melanin levels in the exposure group by the greatest amount as compared to similar concentrations of their other partially hydrogenated counterparts.

Collectively, these results indicate that the effects of compound **1** on the inhibition of melanin levels in cells are not reversible, but after hydrogenation, the effects on melanin levels in cells can be fully reversed; intriguingly, these levels are independent of the degree of hydrogenation.

## 4. Discussion

THC that is formed by catalytic hydrogenation of curcumin has been shown to typically consist of 95.15% THC with other minor components that include 3.40% HHC, 0.94% OHC, and 0.51% tetrahydrodemethoxycurcumin (THDC), as confirmed by LC-MS previously [20]. Our results of compositional analysis of TH-CMC2.24 bear a similar profile to that of THC; we also obtained hexahydro and octahydro derivatives with a higher percentage of the former. Nethinti et al. [13] carried out the hydrogenation of curcumin to yield THC at a higher purity with 10% Pd/C, using diphenyl sulfide catalyst poison and demonstrated that the use of 10% Pd/C was an effective catalyst as it could accelerate the hydrogenation reaction such that <0.1% of the starting compound (curcumin) remained at the end. The authors further reasoned that >0.1% of the starting compound rendered the final hydrogenated compound yellow instead of off-white, which was unacceptable for formulations. Moreover, Nethinti et al. [13] also noted that hexahydrocurcumin and octahydrocurcumin were formed as impurities with THC, similar to our results where we obtained hexahydro and octahydro derivatives of CMC2.24 along with tetrahydro derivative in final products. The method of hydrogenation of curcumin to produce tetrahydrocurcumin and its encapsulation in an excipient has been published and patented by Aurea Biolabs (Kerala, India) and is described elsewhere [15]. Based on this, it was decided to conduct the partial hydrogenation of CMC2.24 in addition to complete hydrogenation. We expected product **5** (TH-CMC2.24) would retain some anti-melanogenic activity of CMC2.24 after the chemical reduction. Thus, it might provide a trade-off between some benefits of skin-lightening with the benefit of a colorless solution. Moreover, we did not expect the partially hydrogenated products **2**, **3**, or **4** to exhibit considerable skin-lightening activity at nontoxic concentrations. However, the novel and unexpected results of this study showed that compound **1** (CMC2.24) retains its anti-melanogenic activity after 1 h of hydrogenation, depending on the concentration. Similarly, the other two partially hydrogenated products (**3** and **4**) also retained appreciable activity to varied degrees without cytotoxicity. Cytotoxicity assays in melanoma cells and keratinocytes showed differential responses for all hydrogenated products. All the hydrogenated products (**2**–**5**) were surprisingly ineffective or cytotoxic in B16F10 cells which was an unexpected result. However, in the case of MNT-1 human melanoma cells, we obtained a concentration-dependent inhibitory effect comparable to parent CMC2.24’s effects at lower concentrations. Compared to the B16F10 cell model, MNT-1 cells are a robust model that better mimics normal human melanocytes due to the shared similarity of the transcriptome [21].

Our previous study [12] demonstrated that CMC2.24 (compound **1**) is a potent CMC that inhibited melanogenesis in HEMn-DP cells, although it had a limited usage window with efficacy only at a single nontoxic concentration of 10 µM (~4 µg/mL or 9.36 µM) and significant cytotoxicity at concentrations > 10 µM. Additionally, CMC2.24 exhibited considerable cytotoxicity to human keratinocytes from the 10 µM concentration onwards. Interestingly, our results of the anti-melanogenic activity of compound **1** in B16F10 cells and HEMn-DP cells align with the results of our previous reports, where it was examined already in B16F10 cells [11] and HEMn-DP cells [12]. Noteworthily, CMC2.24 was included in this study to compare the results of hydrogenated compounds to that of the parent compound to understand the effects better directly. While the hydrogenation of curcumin into THC is not a novel idea as Sabinsa Corp. markets THC (95%), to date, it was not discovered if partial hydrogenation could retain activity without cytotoxicity. In this study, our results showed that the partially hydrogenated products possessed anti-melanogenic activity that was dependent on concentration, also extending the nontoxic range, especially in human keratinocytes and melanocytes, that was not achievable by the parent compound. 

Our results of antioxidant activity evaluated by the DPPH radical scavenging assay showed that none of the samples possess any antioxidant activity (Figure S9). Thus, we confirmed that the hydrogenated products could attenuate melanin production without any antioxidant activity. This imparts a unique marketing edge for cosmetic applications, as it sets it apart from several current skin-lightening agents that diminish melanin production, partly attributable to their high antioxidant activity [22,23,24,25,26]. Notably, compound **1** is an analog of bisdemethoxycurcumin (curcumin lacking two ortho-methoxy groups in the aromatic rings). Bisdemethoxycurcumin has been shown to have low or negligible antioxidant activity [27], and we have shown that CMC2.24 lacked antioxidant activity as evaluated by DPPH radical assay in our previous report [11]. Thus, owing to the lack of antioxidant activity of the parent compound **1** (CMC2.24), the different products (**2**–**5**) formed by hydrogenation reactions also lacked antioxidant activity. Based on the results of the DPPH radical scavenging assay and cellular ROS production in HEMn-DP cells, we concluded that these products do not display any antioxidant capacity. Although it should be emphasized that we only examined intracellular ROS levels (as a key endpoint) that were found to be unchanged, the impact of these hydrogenated products on the activities of antioxidant enzymes such as superoxide dismutase (SOD), catalase, and glutathione peroxidase (GPx) in HEMn-DP cells warrants future investigations. Compounds that can be UV protectant and/or antioxidants are desirable as additives in cosmetic formulations [28,29]. We did not assess if compound **1** or its hydrogenated products (**2**–**5**) might absorb UV radiation or attenuate UV-induced cell death of keratinocytes and/or melanocytes. Hence, whether any of these derivatives can exhibit UV protection for skin needs to be determined in future investigations. Moreover, future experiments to estimate the sun protection factor (SPF) of cosmetic formulations made from these novel derivatives will be interesting.

A comparison of compound **1** and hydrogenated products **2**–**5** at the concentration of 4 µg/mL on cellular melanin levels and tyrosinase activity in HEMn-DP cells (Figure S10) showed an interesting pattern; with increasing time of hydrogenation, the cellular melanin production markedly decreased, while the cellular tyrosinase activity increased after 2 h. In addition, the cell-free tyrosinase activity reached a value similar to the control group at the end of hydrogenation. As the hydrogenated products that diminished melanin levels in DP cells also stimulated tyrosinase activity unexpectedly, we reasoned that this might be correlated to a pro-oxidant milieu that causes the generation of H_2_O_2_, shown previously to augment the intracellular tyrosinase activity in melanocytes [30]. Still, our results on intracellular ROS levels confirmed that the hydrogenated products did not generate ROS species which might have contributed to the increase in the enzyme activity. Overall, the effects of products **4** & **5** on melanin inhibition are not correlated to tyrosinase activity in cell lysates but might occur by a direct effect, as seen in cell-free assays, where these compounds could potently suppress tyrosinase activity catalyzed by monophenolase and diphenolase substrates. Moreover, the anti-melanogenic effects of these hydrogenated products might involve other pathways distal to tyrosinase, such as Tyrosinase-related protein 2 (TYR-2) and TYR-1 [31,32]. Particularly, TYR-1, another critical enzyme involved in the later steps of the melanogenesis cascade, has two zinc ions in its active site instead of copper ions [33]. For example, phenylthiourea, a popular tyrosinase inhibitor, was shown to bind to TYR-1 by hydrophobic interactions that could block the access of the substrate to the active site [34]. As these CMCs have previously been shown to possess a robust zinc-binding capacity, we speculate that the hydrogenated derivatives might also inhibit TYR-1, which should be explored in future studies. 

One exciting result that merits further investigation is that although CMC2.24 did not show any signs of recovery of cellular melanin levels after the 7d period, the extension of duration to evaluate recovery should be explored. If, however, it can be indeed confirmed that CMC2.24 does not lead to any recovery of diminished melanin levels after its removal and prolonged continuation, this might be a beneficial attribute for the clinical (if not cosmetic) use of CMC2.24 as a depigmentation candidate for *vitiligo universalis* for which there is a demand for safe and nontoxic compounds which can induce permanent depigmentation, which does not relapse after discontinuation of the depigmenting regimen [35,36,37]. A rigorous experiment to evaluate this would be worthy of future investigation. One caveat while extrapolating results from single cell culture is that the role of the surrounding cell types is absent, which might have a regulatory effect on the results of recovery experiments. As keratinocytes and melanocytes both exist in a symbiotic manner in the epidermis [38], paracrine factors from keratinocytes that are known to regulate melanogenesis [39,40,41] might be needed to restore melanin levels to baseline. A rigorous study of such a hypothesis might help provide insights into studies of the reversibility of skin depigmenting candidates, which are worthy of further investigation.

The next step would be the evaluation of the hydrogenated CMCs on the 3D organotypic skin tissue model, Melanoderm™, which has remained a popular model for screening pigmentation inhibitors or inducers for cosmetic use [42,43,44]. Because the viability and integrity of Melanoderm models are challenging to maintain beyond the typical treatment duration of 14 d, the assessment of the recovery of melanogenesis by novel skin-lighteners in these models is limited, and there are currently no reports on recovery in a Melanoderm model. Hence, the use of primary human melanocytes to evaluate whether the normal pigmentation of the skin can be reversed upon discontinuing the cosmetic regime of application of the formulation is preferred. In that regard, we have shown the encouraging results of reversible melanin diminution by these hydrogenated derivatives utilizing primary cultures of darkly pigmented cells that are the same cells constituting a Melanoderm tissue.

Despite the efficacy of CMCs, their intense coloration hindered cosmetic formulations, due to which attempts were made to hydrogenate CMCs. CMC2.24 was chemically reduced at different durations to obtain four products (partially and fully hydrogenated). The degree of hydrogenation was a critical factor in obtaining a trade-off between color, efficacy, and nontoxicity. Furthermore, the partially and fully hydrogenated CMC2.24 continued to diminish melanogenesis with far better efficacy compared to parent CMC2.24 with advantages of nontoxicity over a more comprehensive concentration range in both melanocytes and keratinocytes, thus extending the therapeutic use window, which was not possible with CMC2.24. Although we have shown in our previous study [18] that all the hydrogenated derivatives of PC (THC, hexahydrocurcumin; HHC, and octahydrocurcumin; OHC) did not inhibit melanogenesis, validating that hydrogenation of β-diketone of PC results in a loss of its anti-melanogenic capacity, our results from this study suggest that results of our previous study cannot be generalized in light of the findings of this study that reveal that color free TH-CMC2.24, a chemically-modified THC can retain and show a superior anti-melanogenic efficacy for cosmetic uses. 

Compound **1** (CMC2.24) is a triketonic *N*-phenylaminocarbonyl analog of bisdemethoxycurcumin, whose structure consists of an enolic system that shows higher acidic character; this increased acidic character imparts greater capacity to bind zinc and result in markedly higher biological properties including potent anti-melanogenic efficacy, that are not achieved with curcumin [11]. Except for DH-CMC2.24 which retains partial heptadiene moiety (the α-β unsaturated carbonyl group) on one side of the ring, all the other three hydrogenated compounds, TH-CMC2.24, HH-CMC2.24, and OH-CMC2.24 (present in varying % in products), lack the heptadiene moiety that is present in parent compound CMC2.24. Our results of negligible or low cytotoxicity of these hydrogenated products compared to CMC2.24, thus, can be ascribed to the absence of heptadiene moiety in them. This finding agrees with our earlier study, where we showed that the heptadiene moiety of curcumin’s structure is the primary contributor to cytotoxicity, since the hydrogenated compounds THC, HHC, and OHC were nontoxic [18]. Furthermore, despite the lack of heptadiene moiety, the hydrogenated compounds DH-CMC2.24 and TH-CMC2.24 still retain the two β-diketone groups of CMC2.24, while HH-CMC2.24 and OH-CMC2.24 retain one (the other reduced) and no (both reduced) β-diketone groups, respectively. Moreover, in primary melanocytes, we obtained a greater anti-melanogenic potency of product **5** compared to product **4** as at lower concentrations of 4, 8, and 12 µg/mL, melanin levels were further diminished by 18.57%, 20.3%, and 12.09%, respectively. Because product **4** has a nearly identical composition to product **5**, apart from also containing 1% DH-CMC2.24, it can be speculated that the lower efficacy of product **4** is due to trace amounts of DH-CMC2.24. Regarding structure–-activity, the reductive metabolite DH-CMC2.24 still has partial heptadiene moiety compared to TH-CMC2.2,4, which has none [45]. Thus, DH-CMC2.24 will have a greater conjugation leading to higher tautomerism due to the double bonds on one side. Hence, it is likely that higher tautomerism is not beneficial in anti-melanogenic efficacy in primary melanocytes, mainly as the parent compound CMC2.24 exhibits the greatest tautomerism aided by the heptadiene moiety, yet its significant cytotoxicity poses limitations in its use. Further studies to dissect the structure–activity relationships will need the availability of purified metabolites: DH-CMC2.24, HH-CMC2.24, and OH-CMC2.24 to validate this reasoning. Curcumin is known to exhibit keto-enol tautomerism by its β-diketone moiety; in polar solvents, it exists primarily in the enol form, while in non-polar solvents, the keto form predominates [46]. Curcumin can exist in nine possible conformations: one cis-diketo form, two trans-diketo forms, two closed cis-enol forms, one open cis-enol form, and three trans-enol forms [47]. The non-planar THC has been shown to exist in three different conformations in solution: one keto and two enol forms, with the predominance of enol form, which was recently confirmed by spectroscopic analysis [20]. Like THC, TH-CMC2.24 can exist in one keto (**5a**) and two enolic structures, **5b** and **5c** (Figure S11). The presence of other minor compounds as impurities (HH-CMC2.24, OH-CMC2.24) in TH-CMC2.24 and its capacity to exist in keto-enol forms, similar to its progenitor THC, can dictate the biological activities differentially. Moreover, the substituent groups can influence these tautomeric configurations; these different tautomers can also influence biological activities differentially. Interestingly, a recent study reported different biological effects of two stereoisomers of octahydrocurcumin [48]. Isolating these different keto and enolic forms and examining their effects on melanogenesis would be an interesting future study. As in our previous study [18], we reported on the effects of HHC and OHC, hence, whether HH-CMC2.24 and OH-CMC2.24 might have any influence on melanogenesis would also be worth exploring if these can be isolated from the mixture, especially because HH-CMC2.24 that is monoketonic can also exist in enol form in contrast to OH-CMC2.24 (which cannot undergo any enolization as it has no keto group left).

Interestingly, a previous study reported increased % purity of curcumin (99.5%) from curcuminoids by sophisticated crystallization protocols [49]. Currently, it is not known whether the biological activities of 99.99% TH-CMC2.24 (without other minor compounds) might be superior to 95% TH-CMC2.24. Nonetheless, our results of potent inhibition (50% inhibition) of melanogenesis by product **5** at the lowest concentration of 4 µg/mL in primary melanocytes indicated no further need for achieving any better efficacy which might have necessitated a further purification of the product **5**. Moreover, our objective was to compare biological potencies of different hydrogenated mixtures of CMC2.24, which was motivated, in part, due to prior reports that showed that the biological activity of purified curcumin can be markedly different and less potent compared to activity when the curcumin is present in a mixture with other minor compounds/intermediates [50]. For products **2** and **3**, although the purity of TH-CMC2.24 is <92%, this does not limit its application in the cosmetic and nutraceutical sectors where standardized mixtures/extracts are used. This is supported by the fact that prior studies have used commercial-grade curcumin that is not chromatographically pure but has a purity of 60–75% [51,52,53]. As an example, typical commercial-grade curcumin is a curcuminoid mixture consisting of 75–85% curcumin with the presence of 10–20% demethoxycurcumin, and 5% bisdemethoxycurcumin [54]. Moreover, other published studies on tetrahydrocurcumin (THC) have used THC at purity range as the one achieved in our study with products **4** and **5**. As an example, Trivedi et al. [20] used THC that contained 95.15% THC with 0.51% THDMC (tetrahydro demethoxycurcumin), 3.4% HHC, and 0.94% OHC, while Majeed et al. [55] used tetrahydrocurcuminoids which contained 75–85% THC, with 10–20% THDMC, and 2–4.5% THBDMC (tetrahydro bisdemethoxycurcumin). Consequently, the use of mixtures standardized to a specific % of curcumin have been increasingly used, as they are low-cost and easy to scale up for commercial applications and not limited by time-consuming purification steps.

One of the limitations of this study was that the MTS assay employed by us could yield results that are affected due to tetrazolium reduction by the metabolic processes triggered by the presence of the compounds rather than just viable cell numbers. The increased absorbance values in MTS assay by hydrogenated products might occur due to their reducing nature, which has been reported with polyphenolic compounds [56]. Despite this limitation, we confirmed no cytotoxicity for all products after microscopic observation before conducting experiments on melanogenesis. In addition, we normalized our results to total protein contents, which can circumvent any potential confounders of the MTS assay. Further investigations are necessary to evaluate the effects of hydrogenated CMC2.24 (products **4** and **5**) on melanosome export and other steps in the melanogenesis cascade. In this study, the effects of hydrogenated products were only qualitatively examined, and melanosome export was not studied as it was beyond the scope of the study. Nevertheless, since CMC2.24 was shown to suppress melanosome export indirectly by reduced dendricity and attenuation of paracrine melanogenic mediators in our previous report [12], it would be interesting to validate if TH-CMC2.24 might also continue to retain that capacity. We speculate that similar to CMC2.24, TH-CMC2.24 might also suppress the protein levels of endothelin-1, as it retains the metal-binding β-diketone moiety. Moreover, the study of the efficacy of TH-CMC2.24 (alongside CMC2.24 as a reference) on the 3D Melanoderm skin-tissue model would be necessary for future investigations. Lastly, further studies to test excipients encapsulating TH-CMC2.24 are needed.

Melanoma is an aggressive form of skin cancer that originates from the malignant transformation of melanocytes and is associated with a high incidence rate worldwide [57,58,59]. Our results demonstrate the anti-melanogenic effects of partially and fully hydrogenated CMC2.24 for the treatment of skin disorders caused by hyperpigmentation. Based on the encouraging results of high cytotoxicity of hydrogenated products in B16F10 mouse melanoma cells with nontoxicity in normal human melanocytes and human keratinocytes up to a greater concentration range (which was not possible with CMC2.24 due to cytotoxicity), it can be deduced that these products can also have potential as an anti-melanoma therapeutic as they can selectively kill melanoma cells without damaging the normal cells. Additionally, in MNT-1 human melanoma cells, these hydrogenated products attenuated melanin synthesis (although with a lower potency than CMC2.24) instead of causing cell death, which is an advantage as inhibitors of melanogenesis in human melanoma cells have been considered as an adjuvant that can sensitize melanoma cells to the effects of chemotherapy, radiotherapy, or photodynamic therapy [60,61]. As this study focused on using these novel derivatives for skin brightening for cosmetic/cosmeceutical applications, the clinical applications as an anti-melanoma therapeutic were not explicitly explored. Future studies to expand the anti-melanoma potential of these novel derivatives are warranted.

One of the limitations of this study is that an accurate quantification of % purity of the mixtures was not possible, since the authentic standards of DH-CMC2.24, TH-CMC2.24, HH-CMC2.24, and OH-CMC2.24 are not available yet (due to the proprietary nature of the compounds). Due to this, a semi-quantitative estimation of the composition (%) of the four hydrogenated products was conducted by SIM. In addition, the detailed structural characterization (such as C-13 NMR, UV chromatogram) of all the hydrogenated products was beyond the scope of this study, which was primarily focused on the biological evaluation of all the partially and fully hydrogenated products alongside the parent compound on melanogenesis. Future studies to conduct a comprehensive structural characterization in both solid and liquid state for products **4** and **5** in particular, will be necessary.

## 5. Conclusions

Our results provide novel insights into the effects of the role of the degree of hydrogenation of CMC2.24 (lead compound **1**) on melanogenesis, which was dependent on cell type. To the best of our knowledge, this is the first study to show that in primary human melanocytes (HEMn-DP cells), the anti-melanogenic efficacy of the yellow-colored CMC2.24 is retained as early as 1 h after its chemical reduction by hydrogenation; this efficacy is enhanced with longer durations of hydrogenation, with a robust efficacy achieved for the 24 h hydrogenated product **5** at the lowest concentration of 4 µg/mL. A similar potency could be achieved for product **4** at higher concentrations, although interestingly, both differ only by a minor amount of DH-CMC2.24. Our results indicate promise for the use of **4** & **5** for use as a skin-lightener for cosmetic formulations with the triple advantage of lack of any color combined with a potency that is much greater than that of the parent compound CMC2.24 at the lowest concentrations and reversibility of the effects on melanocytes. Additionally, these compounds’ lack of any antioxidant activity suggests that their anti-melanogenic activity is not correlated to any concomitant antioxidant property, which provides a unique marketing edge for cosmetic applications. This, along with the benefits of the easy synthesis method and scale-up of the hydrogenation procedure for these derivatives and the documented higher solubility, stability, and bioavailability of tetrahydrocurcumin, provides further impetus to the incorporation of these derivatives in cosmetic formulations. 

## 6. Patents

The author is listed as an inventor on the patent application describing the use of curcumin analogs for the inhibition of human melanogenesis (U.S. Patent 10,300,000). These patent applications have been fully assigned to the institutions, the Research Foundation of Stony Brook University and ChemMaster International, Inc.

## Data Availability

Data are available from the corresponding author upon reasonable request; some data might not be available due to privacy concerns.

## Supplementary Materials

The following supporting information can be downloaded at: https://www.mdpi.com/article/10.3390/life13061373/s1, Figure S1: 1H-NMR spectra of product **2** in CD_3_OD recorded on 200 MHz NMR instrument; Figure S2: 1H-NMR spectra of product **3** in CD_3_OD recorded on 200 MHz NMR instrument; Figure S3: 1H-NMR spectra of product **4** in CD_3_OD recorded on 200 MHz NMR instrument; Figure S4: 1H-NMR spectra of product **5** in CD_3_OD recorded on 200 MHz NMR instrument; Figure S5: SIM chromatogram of product **2**; Figure S6: SIM chromatogram of product **3** in CD_3_OD recorded on 200 MHz NMR instrument; Figure S7: SIM chromatogram of product **4**; Figure S8: SIM chromatogram of product **5**; Figure S9: DPPH radical scavenging assay for compound **1** and hydrogenated products **2**, **3**, **4**, and **5**; Figure S10: Comparison of the effects of compound **1** and hydrogenated products **2**, **3**, **4**, and **5** at the concentration 4 µg/mL on cellular melanin levels and tyrosinase activity in HEMn-DP cells. Diphenolase activity (cell-free DOPA oxidase) is also shown for comparison; Figure S11: Chemical structures of the three conformations of THCMC2.24: diketo (**5a**) and two enol forms (**5b**, **5c**). The structures were made using the ChemDraw program.
