# Supplementary material for: Novel Hydrogenated Derivatives of Chemically Modified Curcumin CMC2.24 Are Potent Inhibitors of Melanogenesis in an In Vitro Model: Influence of Degree of Hydrogenation
Source: Life (Basel). 2023 Jun 12;13(6):1373. doi: 10.3390/life13061373 (PMC10304319; doi:10.3390/life13061373)
Supplement: Supplementary file 1 [file life-13-01373-s001.zip › life-2383533-supplementary.docx]

**Supplementary Information**

Novel Hydrogenated Derivatives of Chemically-Modified Curcumin CMC2.24 are Potent Inhibitors of Melanogenesis in an in Vitro Model: Influence of Degree of Hydrogenation

Shilpi Goenka^1, 2^*

^1^Department of Biochemistry and Cellular Biology, Stony Brook University, Stony Brook, NY 11794-5215, USA

^2^Department of Biomedical Engineering, Stony Brook University, Stony Brook, NY 11794-5281, USA

* Correspondence: shilp.goenka@gmail.com

**Supplementary Method**

*Antioxidant Activity Assay*

The method used for the determination of antioxidant activity of compounds **1**, **2**, **3**, **4**, and **5** is similar to the method used in our previous study where CMC analogs were studied [*Goenka et al. 2021*]. In a 96-well plate, 20 µL of varying concentrations (12−36 µg/mL) of the compounds were aliquoted followed by the addition of DPPH (2,2-Diphenyl-1-picrylhydrazyl) reagent that was prepared in methanol such that the final DPPH concentration in each well was 60 μM. The microplate was stored at room temperature for 30 minutes, after which the absorbance was recorded at 517 nm and reported for all groups.

**Reference:**

Goenka, S.; Johnson, F.; Simon, S.R. Novel Chemically Modified Curcumin (CMC) Derivatives Inhibit Tyrosinase Activity and Melanin Synthesis in B16F10 Mouse Melanoma Cells. Biomolecules **2021**, 11, 674.

**Supplementary Figures**


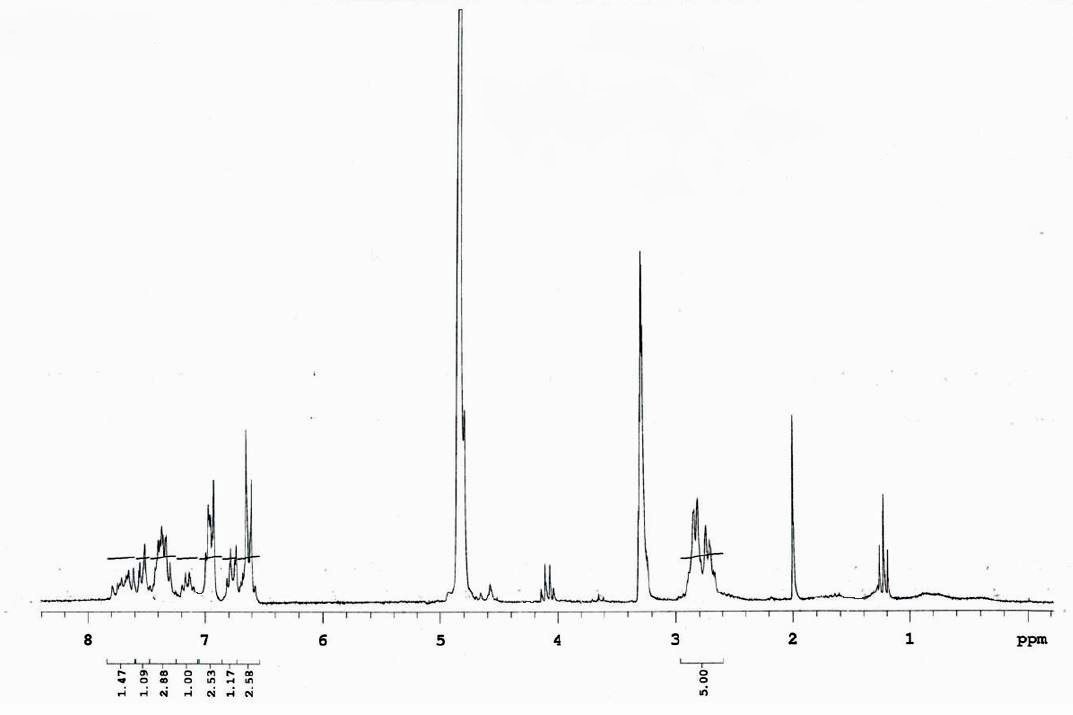


**Figure S1.** 1H-NMR spectra of product **2** in CD_3_OD recorded on 200 MHz NMR instrument**.**


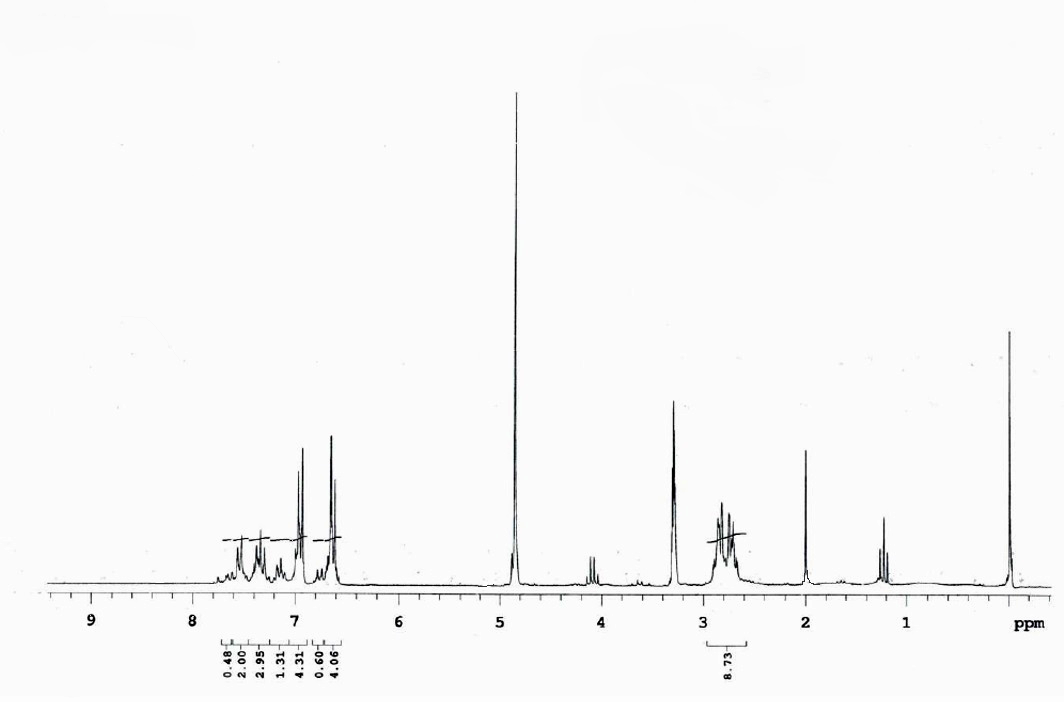


**Figure S2.** 1H-NMR spectra of product **3** in CD_3_OD recorded on 200 MHz NMR instrument**.**


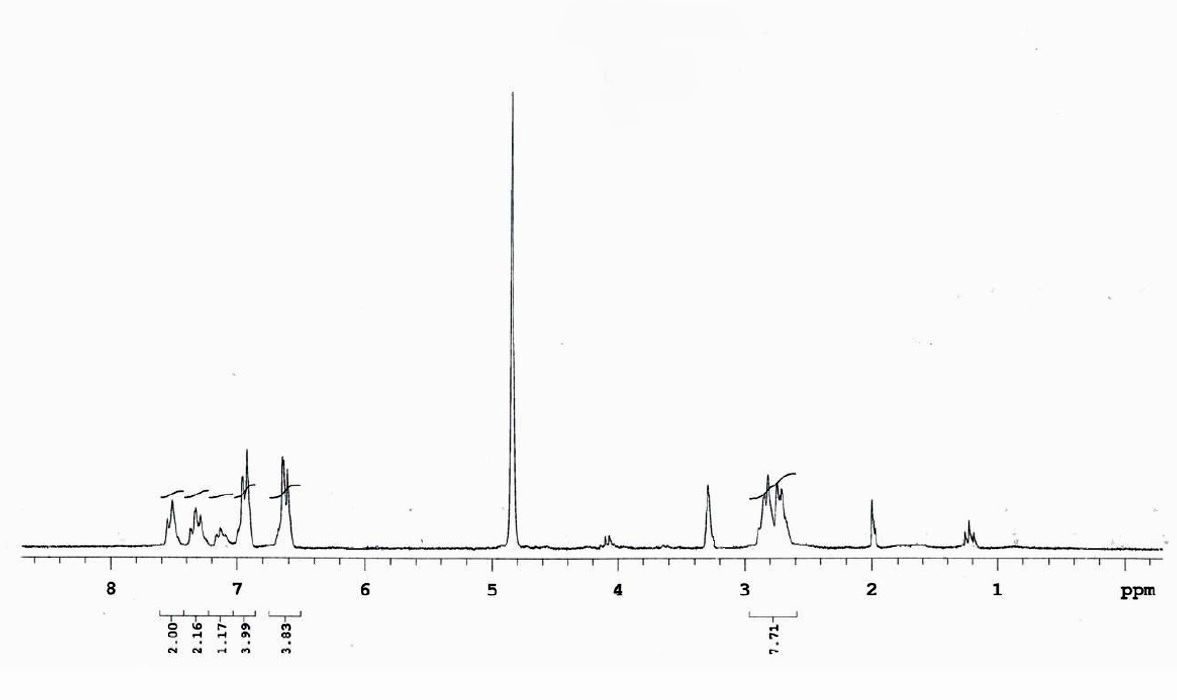


**Figure S3.** 1H-NMR spectra of product **4** in CD_3_OD recorded on 200 MHz NMR instrument**.**

**.**


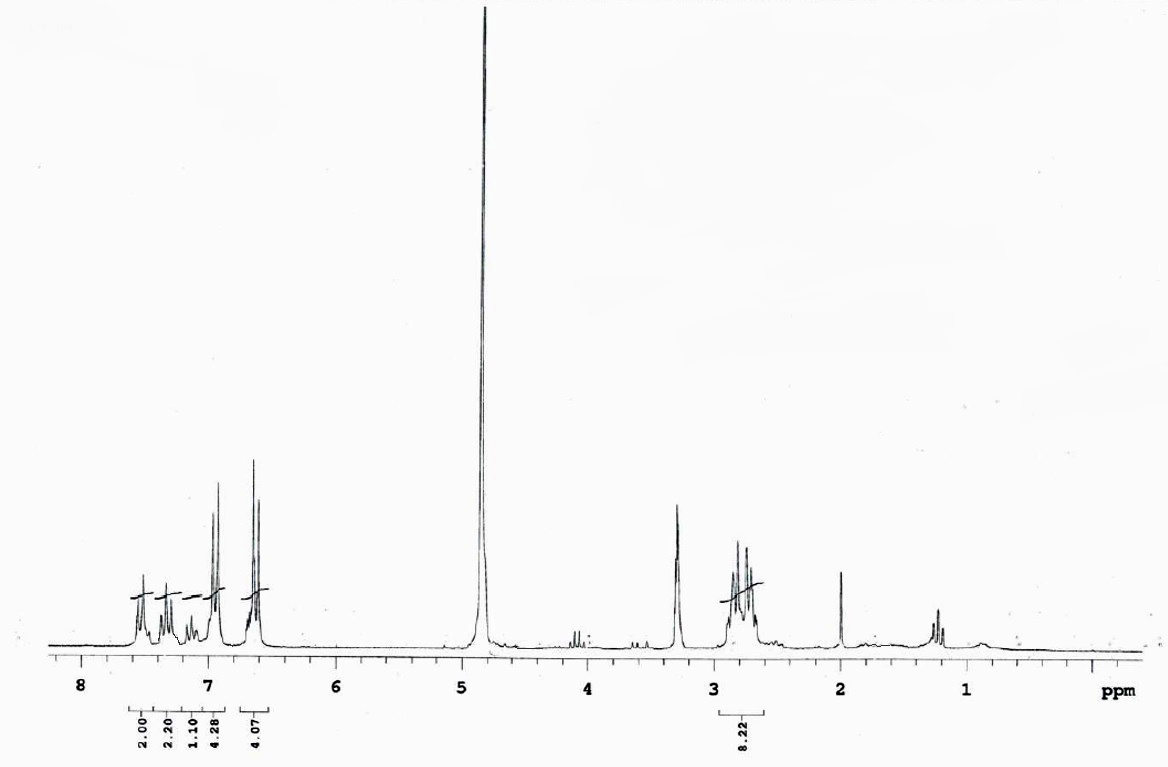


**Figure S4.** 1H NMR spectra of product **5** in CD_3_OD recorded on 200 MHz NMR instrument**.**


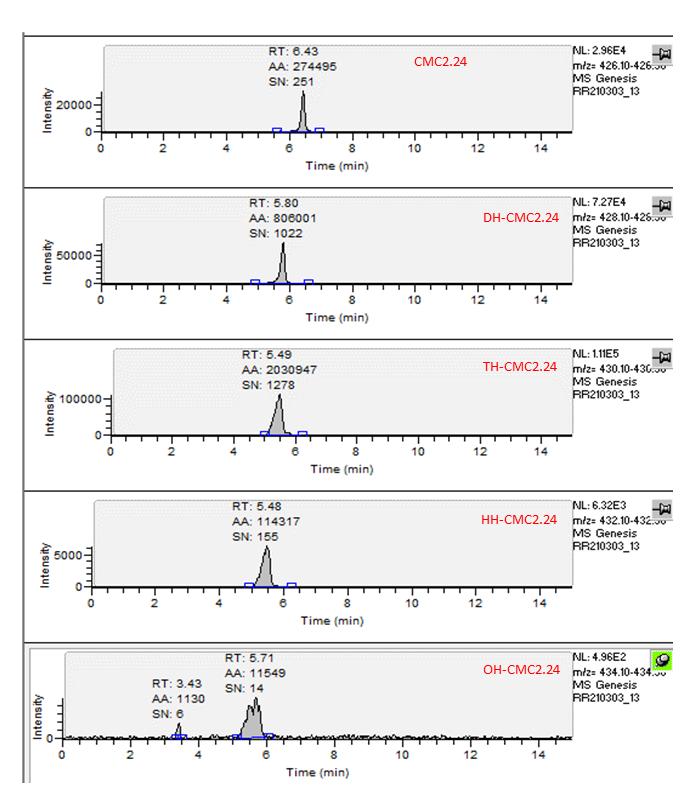


**Figure S5.** SIM chromatogram of product **2.**


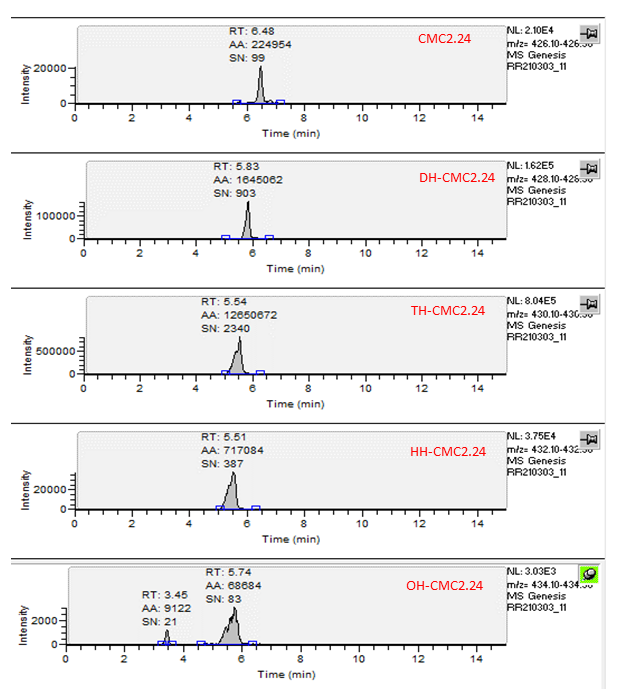


**Figure S6.** SIM chromatogram of product **3.**


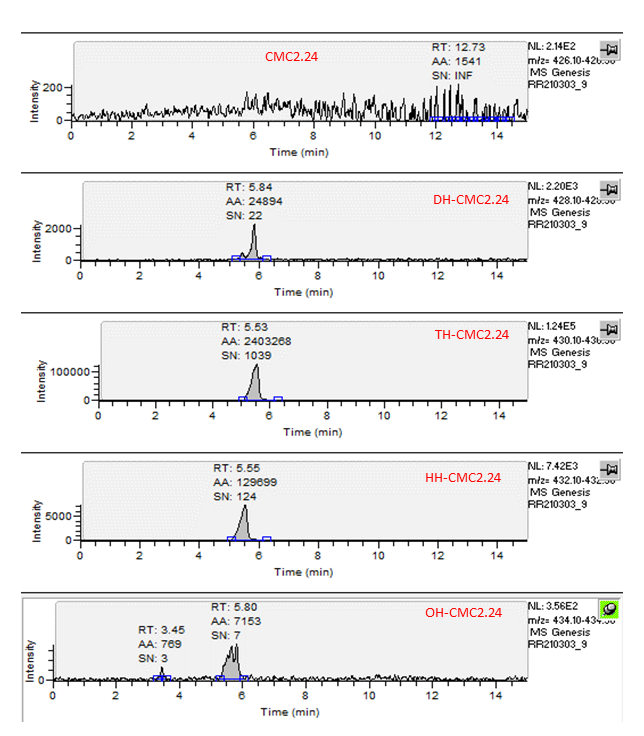


**Figure S7.** SIM chromatogram of product **4.**


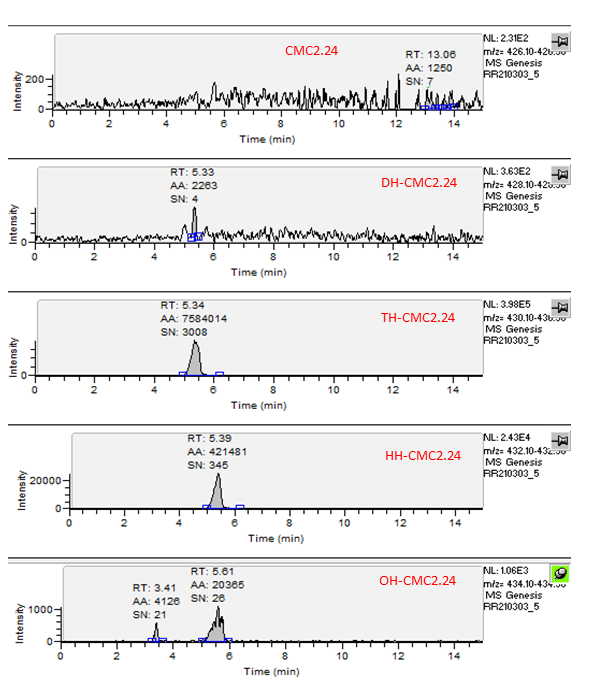


**Figure S8.** SIM chromatogram of product **5.**


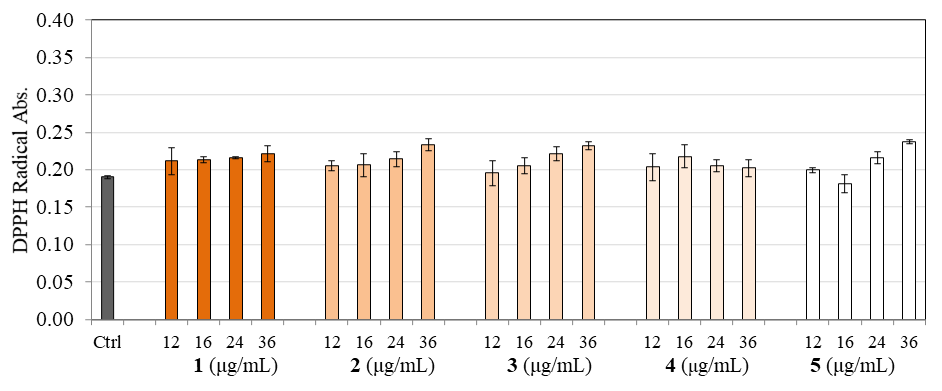


**Figure S9.** DPPH radical scavenging assay for compound **1** and hydrogenated products **2**, **3**, **4**, and **5**.


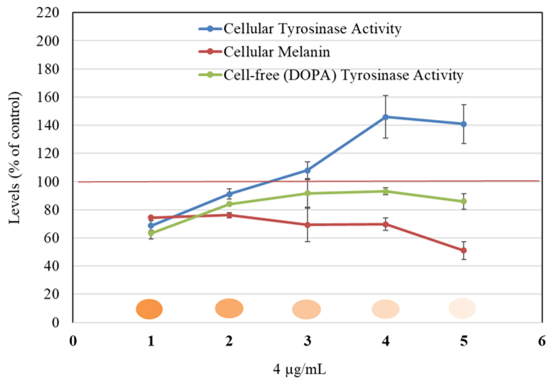


**Figure S10.**  Comparison of the effects of compound **1** and hydrogenated products **2**, **3**, **4**, and **5** at the concentration 4 µg/mL on cellular melanin levels and tyrosinase activity in HEMn-DP cells. Diphenolase activity (cell-free DOPA oxidase) is also shown for comparison.


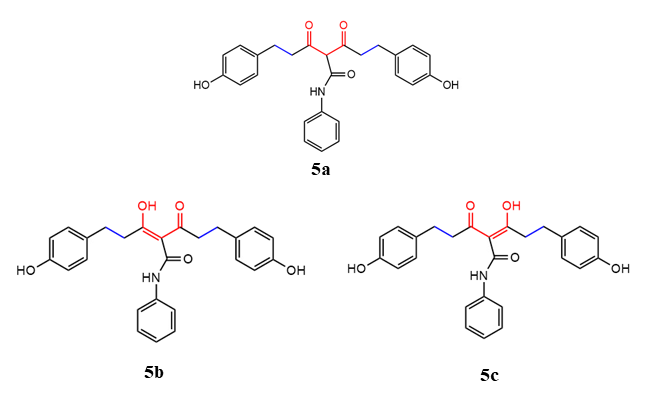


**Figure S11.**  Chemical structures of the three conformations of THCMC2.24: diketo (**5a**) and two enol forms (**5b**, **5c**). The structures were made using ChemDraw program.
